# Radar Cross Section Near-Field to Far-Field Prediction for Isotropic-Point Scattering Target Based on Regression Estimation

**DOI:** 10.3390/s20216023

**Published:** 2020-10-23

**Authors:** Yang Liu, Weidong Hu, Wenlong Zhang, Jianhang Sun, Baige Xing, Leo Ligthart

**Affiliations:** 1 School of Information and Electronics, Beijing Institute of Technology, Beijing 100081, China; 3120160335@bit.edu.cn (Y.L.); 3120180713@bit.edu.cn (B.X.); 2 Department of Computing, The Hong Kong Polytechnic University, 11 Yuk Choi Rd, Hung Hom, Hong Kong 999077, China; wenlong.zhang@connect.polyu.hk; 3 China North Industries Corp., Beijing 100053, China; sunjh@norinco.com; 4 Faculty of Electrical Engineering, Delft University of Technology, 2628 CN Delft, The Netherlands; leoligthart@kpnmail.nl

**Keywords:** near-field to far-field transformation (NFFFT), neural network, nonlinear regression, radar cross section (RCS) measurement, regression analysis

## Abstract

Radar cross section near-field to far-field transformation (NFFFT) is a well-established methodology. Due to the testing range constraints, the measured data are mostly near-field. Existing methods employ electromagnetic theory to transform near-field data into the far-field radar cross section, which is time-consuming in data processing. This paper proposes a flexible framework, named Neural Networks Near-Field to Far-Filed Transformation (NN-NFFFT). Unlike the conventional fixed-parameter model, the near-field RCS to far-field RCS transformation process is viewed as a nonlinear regression problem that can be solved by our fast and flexible neural network. The framework includes three stages: Near-Field and Far-field dataset generation, regression estimator training, and far-field data prediction. In our framework, the Radar cross section prior information is incorporated in the Near-Field and Far-field dataset generated by a group of point-scattering targets. A lightweight neural network is then used as a regression estimator to predict the far-field RCS from the near-field RCS observation. For the target with a small RCS, the proposed method also has less data acquisition time. Numerical examples and extensive experiments demonstrate that the proposed method can take less processing time to achieve comparable accuracy. Besides, the proposed framework can employ prior information about the real scenario to improve performance further.

## 1. Introduction

**Figure 1:**
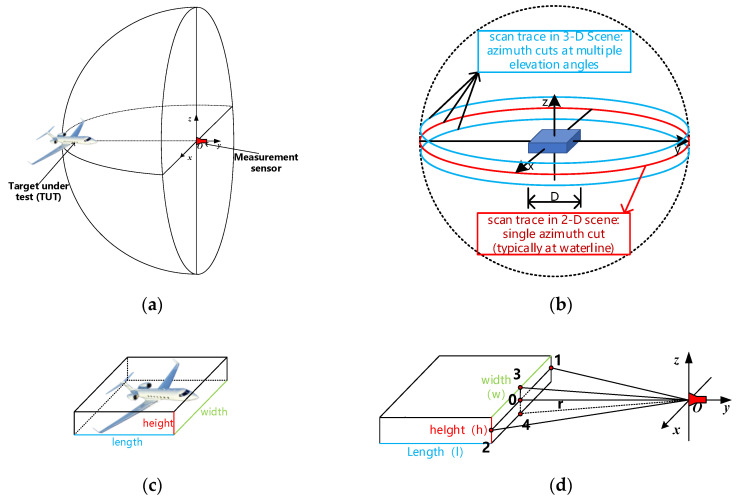
Schematic diagram for radar cross section measurement: (**a**) Incident spherical wave in the target under test (TUT); (**b**) Near-field scattering data collection geometries; (**c**) The geometry of the TUT; (**d**) Incident wave analysis of the TUT.

The radar cross section (RCS) of an object is a fictitious area that describes the intensity of the reflected wave in the radar [1]. The RCS definition requires that the target be located at infinity distance, which makes sure that a plane wave illuminates the target. In general, the target and measurement sensors are always situated at a finite distance apart. Thus, the incident wave is spherical, as shown in Figure 1a. In order to measure RCS with an acceptable error 1 dB or less [1], the target must be at distances greater than $2d^{2}/\lambda$, where d is the linear size of the target under test (TUT) and λ is the wavelength [2]. This is the far-field criterion. It implies that, with the frequency increasing or the target expanding, the far-field range will be too extensive to permit RCS direct measurements.

As shown in Figure 1c,d, if the measurement distance r does not meet the far-field criterion both in the horizontal and vertical directions—i.e., $r<2w^{2}/\lambda$ & $r<2h^{2}/\lambda$—the incident wave is spherical. While if the measurement distance r meets the far-field criterion in the vertical direction but not in the horizontal direction—i.e., $r>2w^{2}/\lambda$ & $r<2h^{2}/\lambda$—the phase displacement between point 3 and point 0 is small enough (less than π/8) [1]. Under this condition, the wave can be treated as a cylindrical wave.

If a TUT is measured in the range which does not meet the far-field criterion, the scattered field measured by sensors is called near-field. RCS calculated by near-field has an unacceptable error and needs to be transformed into far-field RCS. This process is named as the near-field to far-field transformation (NFFFT).

There are many common approaches to implement NFFFT based on electromagnetic theory. Most of them are called “image-based” techniques. That is because they need to make broadband measurements on the target. This “image-based” method can be summarized in two ways. The first kind evaluates the inverse synthetic aperture radar (ISAR) images in near-field and calculates RCS directly, as in [3,4,5,6]. The accuracy of the first kind depends on the imaging process. The measured errors lead to corresponding errors in the reconstructed far-field RCS [3]. The second kind, based on the Hankel function, calculates RCS without the image reconstruction process but still needs a broadband measurement, as in [7,8,9,10]. However, this method introduces fluctuations in the angle domain [9]. While spatial filtering techniques may suppress the fluctuations, they introduce a significant loss of angle resolution [11]. All of the “image-based” techniques require a broadband measurement in a wide-angle. Besides the “image-based” approach, NFFFT is also implemented by plane-wave expansion in [12,13]. In this kind of method, the exact shape of the object is needed. The accuracy of the algorithm depends on the sampling position and the discretization. The methods mentioned above reveal the relationship between near-field and far-field, while they both require a long time in data acquisition and signal processing.

In this paper, firstly, based on the Swerling Case I Model, point-scattering targets are used to simulate Near-field and Far-field data. The training samples can be easily acquired by measurements and simulations. Secondly, a new framework, called Neural Networks Near-Field to Far-Filed Transformation (NN-NFFFT), is proposed to predict the far-field RCS for isotropic-point scattering targets. The proposed method views NFFFT as a regression problem and electromagnetic scattering characteristics are integrated into the dataset as a priori knowledge. In contrast, the conventional method is a fixed parameter model.

Specifically, NN-NFFFT introduces a lightweight neural network to predict far-field RCS from the single frequency point near-field RCS data. Compared with the traditional method, the proposed NN-NFFFT can achieve more than ten times faster than the “Image-Based” NFFFT while maintaining comparable accuracy. The well-trained estimator can be used as a real-time mapping function between near-field RCS and far-field RCS. This process is shown in the simulation section.

The result shows that the far-field RCS can be predicted by the neural network with prior information efficiently. The complex calculations and the accuracy of the framework are only included in the training process. By using a large amount of data, the framework can be trained to meet the error requirement.

Furthermore, the antennas’ radiation patterns and the bistatic measurement can be completely incorporated in the training process with no additional complication. The sampling limitation introduced in [10] can also be ignored due to the training samples. The experiment result shows that, for the target, which has a small RCS, the method maintains good accuracy with less operation time.

## 2. Theory

### 2.1. The Relationship between Near-Field and Far-Field

RCS is defined as

(1) $$\sigma =4\pi \underset{R\to \infty }{\lim}R^{2}\frac{{\left|E_{s}\right|}^{2}}{{\left|E_{i}\right|}^{2}}$$

where $E_{s}$ is a component of the scattered electric field at an observation point, and $E_{i}$ is a component of the incident electric field at the target position.

To implement NFFFT, it requires that the target must satisfy the scalar SAR “reflectivity density” model. The reflectivity distribution ρ(r′) does not change with the look angle [6].

**Figure 2:**
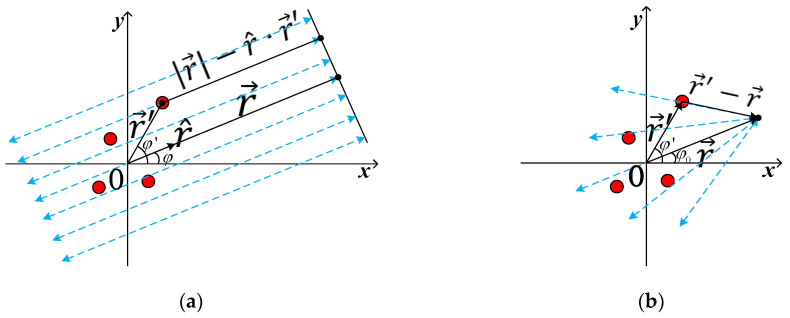
Two-dimensional far-field and near-field condition: (**a**) Far-field condition; (**b**) Near-field condition.

When a spherical wave illumines a target, the whole measurement process is a 3D scene, as shown in Figure 1b through the blue and red lines. If the target is flat enough, as mentioned above, it is approximately illumined by a cylindrical wave. This situation is common in RCS measurement because both aircrafts and vehicles are flat. In this condition, measuring sensors trace is shown in Figure 1b through the red line. The target model can be described in the *x*-*y* plane, as shown in Figure 2.

As shown in Figure 2a, the target is illuminated by a plane wave under the far-field condition. The monostatic scattered field at the receiver sensor is given by [5]

(2) $$S_{FF}\left(\hat{r},k\right)=C_{\mathrm{FF}}\int_{V}\rho \left(r\prime \right)e^{-j2k\left(\left|r\right|-\hat{r}\cdot r\prime \right)}dr\prime$$

where ρ(r′) is the TUT’s reflectivity distribution. $\hat{r}$ is a unit vector that represents the look angle. k stands for wavenumber, which is equal to 2πf/c. r stands for the measurement range. r′ indicates the location of the target. $C_{FF}$ is a constant that depends on measurement system’s parameters, which can be removed by calibration.

In the two-dimensional near-field scene, as shown in Figure 2b, the scalar Green’s function in free space can be expressed as $G_{0}\left(r,r\prime \right)=\exp\left(-jk\left|r-r\prime \right|\right)/4\pi \left|r-r\prime \right|$, Every scattering point on the target can be regarded as a reradiating source. Thus, the new two-way Green’s function is $G\left(r,r\prime \right)=\exp\left(-j2k\left|r-r\prime \right|\right)/{\left(4\pi \left|r-r\prime \right|\right)}^{2}$ [8]. In this condition, the monostatic scattered field at the receiver point is given by

(3) $$S_{NF}\left(r,k\right)=C_{NF}\int_{V}\rho \left(r\prime \right)\frac{e^{-j2k\left|r-r\prime \right|}}{{\left|r-r\prime \right|}^{2}}dr\prime$$

where $C_{NF}$ is a constant that depends on the parameters of the system and can be removed by calibration. r stands for the measurement range and r′ indicates the location of the target. Equation (3) can be rearranged to the format including Green’s function of 2-D free space [8]

(4) SNF(r,k)=∫V2kρ(r′)|r−r′|32e−j2k|r−r′|2k|r−r′|dr′

As shown in Figure 2b, the 2-D condition is discussed. Considering Hankel addition theory, Green’s function of 2-D free space can be expressed as

(5) $$\frac{e^{-j2k\left|r-r\prime \right|}}{\sqrt{2k\left|r-r\prime \right|}}=H_{0}^{\left(2\right)}\left(2k\left|r-r\prime \right|\right)=\sum_{n=-\infty }^{\infty }H_{n}^{\left(2\right)}\left(2kr\right)J_{n}\left(2kr\prime \right)e^{jn\left(\varphi_{0}-\varphi \prime \right)}$$

where $H_{n}^{\left(2\right)}$ is the Hankel function of the second kind and $J_{n}$ is the Bessel function. φ stands for the far-field look angle while $\varphi_{0}$ is the near-field look angle. φ′ is the angle of the point on the TUT. Thus Equation (3) can be changed as

(6) SNF(r,k)=∫V2kρ(r′)|r−r′|32∑n=−∞∞Hn(2)(2kr)Jn(2kr′)ejn(φ0−φ′)dr′

Exchanging the position of integration and summation, Equation (6) becomes

(7) SNF(r,k)=∑n=−∞∞{Hn(2)(2kr)ejnφ0[∫V2kρ(r′)|r−r′|32Jn(2kr′)e−jnφ′dr′]}

While in the far-field condition, the measurement distance **r** in Equation (7) is infinity—i.e., r→∞. Then Equation (7) turns into the far-field monostatic scattered field given by

(8) SFF(r,k)=limr→∞SNF(r,k)=limr→∞∑n=−∞∞{Hn(2)(2kr)ejnφ[∫V2kρ(r′)|r−r′|32Jn(2kr′)e−jnφ′dr′]}

The relationship between the TUT and the measurement sensor turns from Figure 2b to Figure 2a. Furthermore, the representation of the look angle is also changed from $\varphi_{0}$ to φ. Due to $\left|r\right|\gg \left|r\prime_{\max}\right|$ ($r\prime_{\max}$ represents for the maximum size of the TUT) in the far-field condition, |r−r′| can be approximated as |r| or r without any error. The following can be derived

(9) $$S_{FF}\left(r,k\right)=\sqrt{\frac{2k}{r^{3}}}\sum_{n=-\infty }^{\infty }\{H_{n}^{\left(2\right)}\left(2kr\right)e^{jn\varphi }[\int_{V}\rho \left(r\prime \right)J_{n}\left(2kr\prime \right)e^{-jn\varphi \prime }dr\prime ]\}$$

Note $\int \rho \left(r^{\prime }\right)J_{n}\left(2kr^{\prime }\right)\exp\left(-jn\varphi^{\prime }\right)dr^{\prime }=S_{n}^{2k}$ is the generalized Fourier series of the target image, an inherent scattering characteristic of the target and not related to measurement distance.

Equation (9) can be changed as

(10) $$S_{FF}\left(r,k\right)=\sqrt{\frac{2k}{r^{3}}}\sum_{n=-\infty }^{\infty }S_{n}^{2k}H_{n}^{\left(2\right)}\left(2kr\right)e^{jn\varphi }$$

In the far field range—i.e., 2kr→∞—using the large argument approximation theory in Hankel function [14], Equation (10) becomes

(11) $$S_{FF}\left(r,k\right)\approx \frac{1}{r^{2}}\sqrt{\frac{2}{\pi }}e^{-j\left(2kr-\frac{\pi }{4}\right)}\sum_{n=-\infty }^{\infty }S_{n}^{2k}e^{jn\left(\varphi +\frac{\pi }{2}\right)}$$

As mentioned before, the generalized Fourier series of the target image $S_{n}^{2k}$ is not related to measurement distance. Near-field data can also represent it.

In [7], weighted near-field data $U_{NF}$ are created by Fourier Transform:

(12) $$U_{NF}=\mathrm{FFT}\left({\left|r-r\prime \right|}^{\frac{3}{2}}\cdot \mathrm{IFFT}\left(S_{NF}\right)\right)=\int_{V}\sqrt{2k}\rho \left(r\prime \right)\frac{e^{-j2k\left|r-r\prime \right|}}{\sqrt{2k\left|r-r\prime \right|}}dr\prime$$

Combining Equations (5), (12) and the definition of $S_{n}^{2k}$, $U_{NF}$ can be changed as

(13) $$U_{NF}\left(r,k\right)=\sqrt{2k}\sum_{n=-\infty }^{\infty }S_{n}^{2k}H_{n}^{\left(2\right)}\left(2kr\right)e^{jn\varphi_{0}}$$

$S_{n}^{2k}$ can be acquired by applying Fourier transform to near-field data $U_{NF}\left(r,k\right)$, given by

(14) $$S_{n}^{2k}=\sqrt{\frac{1}{2k}}\frac{\int U_{NF}\left(r,k\right)e^{-jn\varphi_{0}}d\varphi_{0}}{H_{n}^{\left(2\right)}\left(2kr\right)}$$

Ignore the coefficient, which can be determined by calibration. Combining (11) and (14), the relationship between the near-field data $U_{NF}\left(r,k\right)$ and the far-field data $S_{FF}\left(r,k\right)$ can be derived as:

(15) $$S_{FF}\left(r,k\right)=\int U_{NF}\left(r,k\right)\sum_{n=-\infty }^{\infty }\frac{j^{n}e^{jn\left(\varphi -\varphi_{0}\right)}}{H_{n}^{\left(2\right)}\left(2kr\right)}d\varphi_{0}=U_{NF}\left(r,k\right)*\omega \left(\varphi_{0}\right)$$

where $\omega \left(\varphi_{0}\right)=\overset{N_{0}}{\underset{-N_{0}}{\sum }}j^{n}\exp\left(jn\varphi_{0}\right)/H_{n}^{\left(2\right)}\left(2kr\right)$ stands for the NFFFT process.

According to [6], the RCS of the TUT is defined as

(16) $$\sigma \left(r,k\right)=C{\left|S_{FF}\left(r,k\right)\right|}^{2}$$

where *C* is a constant determined by calibration. Thus, the near-field RCS is defined as

(17) $$\sigma_{NF}\left(r,k\right)=C{\left|S_{NF}\left(r,k\right)\right|}^{2}$$

Our framework focuses on predicting the far-field RCS from the near-field RCS. Unlike the conventional approach, this process is nonlinear.

### 2.2. Regression Estimation

Equations (12) and (15) reveal the relationship between near-field and far-field measurement data in theory. This process is liner, ignoring the multi-reflection [12]. Combining the NFFFT process with the RCS definition in Equations (16) and (17), the radar cross section near-field to far-field transformation is nonlinear.

In our framework, the NFFFT problem is defined as a nonlinear regression problem. From the Bayesian inference framework [15], the maximum a posteriori (MAP) finds the optimal RCS σ′

(18) $$\sigma \prime =\arg\underset{\sigma }{\max}p\left(\sigma |\sigma_{NF},\xi \right)p\left(\sigma \right)$$

where σ is true far-field RCS while σ′ is the optimal one. ξ stands for the framework’s parameter to be optimized. p(σ) corresponds to the prior distribution of σ. Equation (18) can be changed into logarithmic form to find the most suitable parameter:

(19) $$\sigma \prime =\arg\underset{\sigma }{\max}\log p\left(\sigma |\sigma_{NF},\xi \right)+\log p\left(\sigma \right)$$

Equation (19) can be further transformed to represent the loss function:

(20) $$\sigma \prime =\arg\underset{\sigma }{\min}\frac{1}{2}{\left\|\sigma_{NF}-\xi \cdot \sigma \right\|}^{2}+\lambda \phi \left(\sigma \right)$$

where σ′ can be obtained by minimizing the $0.5{\left\|\sigma_{NF}-\xi \cdot \sigma \right\|}^{2}$. A regularization term ϕ(σ) can restrict the model’s learning ability on the dataset so that the trained model has a stronger generalization ability for the new data. The larger the value of parameter λ, the greater the penalty for the model.

**Figure 3:**
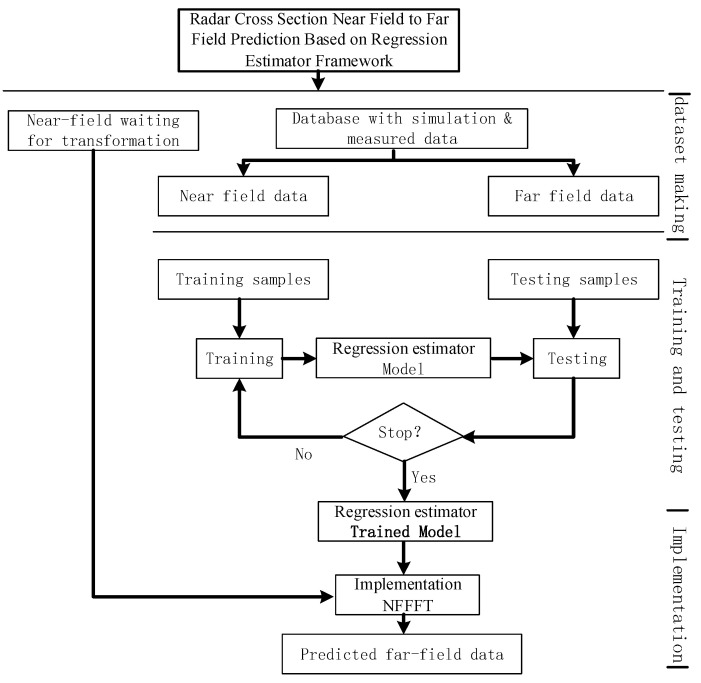
Near-field to far-field transformation framework flow chart.

Based on (20), a regression estimator can be used to approach the mapping relationship between near-field RCS and far-field RCS. In order to approximate the whole process, large amounts of data pairs are needed. This process is called making a dataset. Then, the data pairs are used to train a specific estimator model using a nonlinear regression approach. If the trained estimator model meets the error requirements, it can be used as a prediction system for the NFFFT. This process is shown in Figure 3.

### 2.3. Neural Network

In this paper, the regression estimator is implemented by a supervised neural network (NN).

#### 2.3.1. Fundamental Theory

A feed-forward artificial neural network used sigmoid or Gaussian activation function is a universal estimator [16]. It has already been proposed as an efficient tool to implement antenna radiation pattern NFFFT in [17].

In our framework, the L2 parameter norm penalty is used in Equation (20). The L2 parameter norm penalty is defined as

(21) $$\phi \left(\sigma \right)=\frac{1}{2}{\left\|\xi \right\|}_{2}^{2}$$

L2 regularization causes the NN to “perceive” the input data as having higher variance, which makes it shrink the weights on features whose covariance with the output target is low compared to this added variance [18].

**Figure 4:**
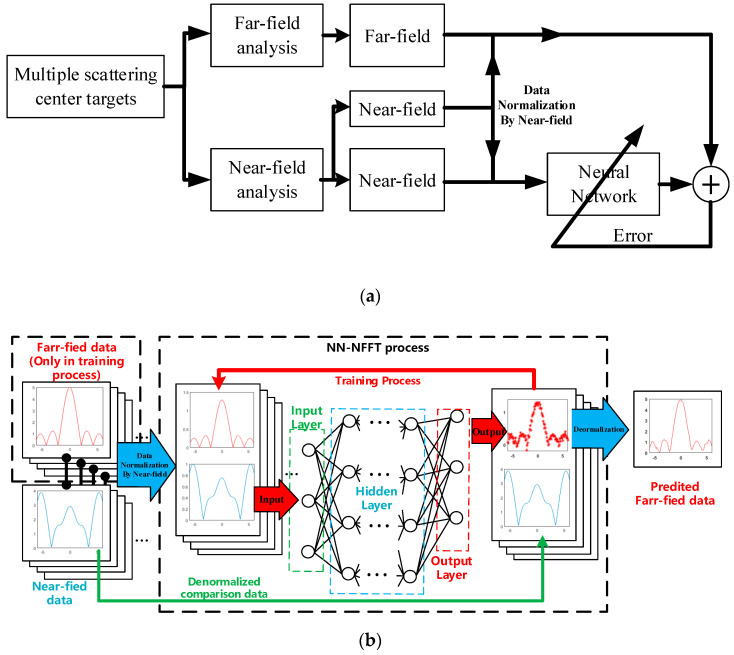
Neural network flow chart (**a**) NN-NFFFT training scheme (**b**) the structure of NN-NFFFT framework.

A set of random isotropic scattering center distributions is generated to calculate near-field RCS over the angle of measurement and the same angle of far-field RCS. Before importing to the neural network, the maximum value of the input near-field RCS data is used to normalize the near-field and the corresponding far-field data to obtain a data pair. Then they are used to train the neural network. During the training process, according to Equation (20), the main aim of training is to optimize the NN model’s weight parameter to achieve an appropriate transformation network. This process is shown in Figure 4a.

During the implementation process, the input near-field data needs to be normalized first, and then it enters into the NN to predict the far-field RCS. The last step before the output is that the same value is used to denormalize the predicted RCS data. The structure of the NN-NFFFT framework is shown in Figure 4b.

Besides the neural network, convolutional neural network [19,20], deep residual network [21,22], generative adversarial networks [23] or the other deep learning models can also be used as a regression estimator.

#### 2.3.2. Neural Network Structure

The network architecture of the proposed NN has five layers. In this NN structure, three hidden layers are used: two layers have 256 neurons connected to the input, and output layers and one layer in the middle has 512 neurons. The sigmoid function is chosen as an activation function and the regularization parameter λ = 0.05.

#### 2.3.3. Priori Information

A large number of prior data pairs are needed to train the NN model. The data must have a similar probability distribution to the TUT.

As mentioned in [24], multiple scattering center targets can directly define the monostatic scattering field at any point. Once the scattering center distribution is known, the near-field and far-field data can be simulated as prior data pairs. What is more, the training dataset can also use measured data, which is easily accessible in the RCS test field.

It is of vital importance to find an appropriate modeling method. The most related modeling work is in fluctuating target detection [25]. The radar signal fluctuation is mainly caused by look angle changing. Marcum and Swerling have presented several fluctuating models to investigate the signal fluctuation distribution, which are summarized in [25]. Those models contain most of the radar detection targets.

Modeling in this paper is inspired by the Swerling Case I Model [26], which is commonly used to represent small jet aircraft in the front view. The Swerling Case I Model is defined as a target that can be represented as several independently fluctuating reflectors of approximately equal echoing area, even if the number of reflectors is as small as four or five.

**Figure 5:**
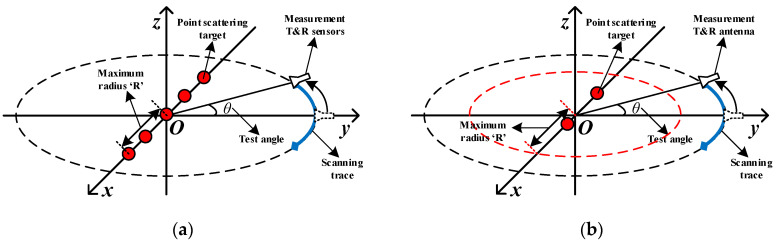
Point scattering model and near-field measurement setting (**a**) an example of 5-points model (**b**) an example of a randomly placed 2-points model.

A group of centrosymmetric point-scattering targets is used as a prior information. The target consists of three to five equal scattering centers that are evenly located on the *x*-axis. The maximum radius of it is R. The monostatic test antenna is located at a distance L away from the origin. Figure 5a shows an example of our model that is conforming to the Swerling Case I Model.

Furthermore, the experiment demonstrates that the neural network is a potential method to achieve NFFFT, based on this model. For a specific scatterer, the far-field RCS can be predicted by a neural network with prior information efficiently.

#### 2.3.4. Evaluation

In order to assess the error in the results, in [12], the root-mean-square error (RMSE) is used. RMSE is defined as

(22) $$\mathrm{RMSE}=\sqrt{\frac{1}{N_{\theta }}\sum_{n=1}^{N_{\theta }}{\left|\sigma_{meas}\left(n\right)-\sigma_{Extr.}\left(n\right)\right|}^{2}}$$

where $\sigma_{meas}\left(n\right)$ (units $m^{2}$) is the RCS output by the neural network. $\sigma_{Extr}\left(n\right)$ (units $m^{2}$) is the far-field RCS defined by the target and *n* is the sampling point in the angle domain. The unit of the RMSE is $m^{2}$ and it can be changed in to dBsm. RMSE stands for the absolute error in the whole testing angle.

## 3. Numerical Analysis

### 3.1. Setting

In the implementation of the framework, the test angle *θ* is from −6° to 6° and has 121 sampling points. It takes into account the cost of data acquisition and the limitations of “image-based” NFFFT on the test angle [10]. The radius R of the target is limited to 3λ to 8λ. In this condition, the far-field range is 72λ to 512λ, while the measurement distance L is 33λ.

Considering the balance between accuracy and training speed, the selection process of the training sample has the following steps.

Firstly, we create three different datasets. They have 3 × 66, 3 × 122 and 3 × 244 samples, respectively. The more samples we use, the closer the test error is to the training error.

Then, 45 samples are also randomly selected in the range of R to test the trained neural network framework. They are independent of the training samples and do not participate in the training process. There are 15 samples for the three, four, and five scattering centers, respectively. The RCS of each point is 0 dBsm (1 $m^{2}$). They are used to verify the error of the new data and are enough to demonstrate the framework’s applicability.

**Figure 6:**
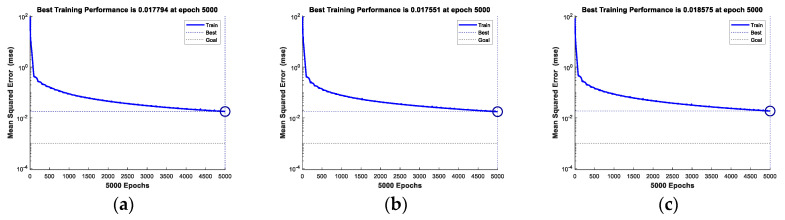

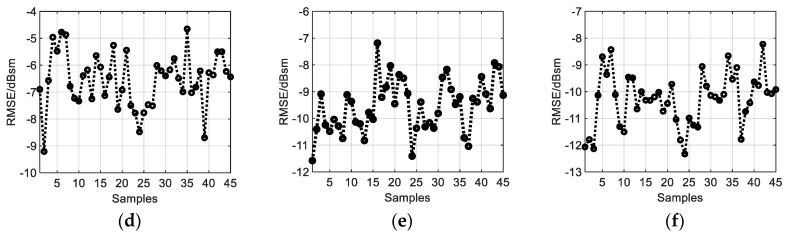
The training and testing result of three different datasets (**a**) training error for 3 × 66 samples (**b**) training error for 3 × 122 samples (**c**) training error for 3 × 244 samples (**d**) testing RMSE for 3 × 66 samples (**e**) testing RMSE for 3 × 122 samples (**f**) testing RMSE for 3 × 244 samples.

The iteration of the training is 5000 times. The error-iteration of the models is shown in Figure 6a–c. After 5000 iterations, the training set’s error is lower than −16 dB, and the training error between different datasets is not significant. The testing error is shown in Figure 6d–f. The training time of different datasets is 180, 275 and 380 s. Comparing the balance between accuracy and training speed, we selected 366 training samples. They are randomly picked with the radius limitation. The training samples are composed of three equal parts. There are 112 samples for the three, four, and five scattering centers, respectively.

### 3.2. Comparison

In order to compare with “image-based” NFFFT in [10], a three-point scattering target that meets the requirements in Figure 5a is used to define the wideband monostatic scattering field at the same angle. The center frequency of them is the same as the NN-NFFFT framework, while the relative bandwidth is 40%. The maximum radius R is 5.2 λ.

As mentioned in [10], the accuracy of “Image-Based” NFFFT is relative to the sample points in the frequency domain and the truncation number in Equation (15). The truncation number $n_{max}$ is related to the minimum diameter of the target D (D = 2R) and is restricted to be greater than kD+10 [9], where *k* is the wavenumber. If the measurement distance and the test angle is decided, the error and runtime of NFFFT are only related to the frequency sampling points.

In this paper, eight groups of different frequency sampling points (401, 201, 101, 81, 51, 21, 11, 5) data are generated. The wideband near-field data are used in Equation (15) to implement “Image-Based” NFFFT to compare with the NN-NFFFT framework.

### 3.3. Performance

**Figure 7:**
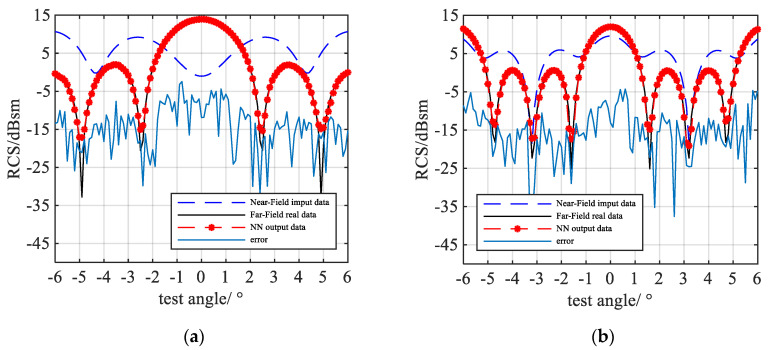

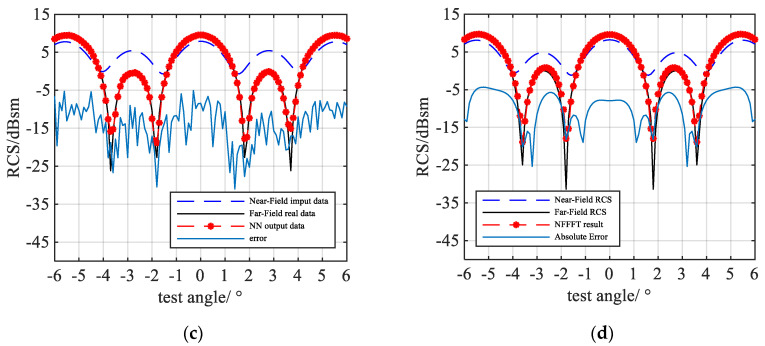
Simulation result for different test samples: (**a**) 5-point target R = 5.47λ NN-NFFFT result (**b**) 4-point target R = 6.8λ NN-NFFFT result (**c**) 3-point target R = 5.2λ NN-NFFFT result (**d**) 3-point target R = 5.2λ “image-based” NFFFT result with 401 frequency points.

Four of the NFFFT results are shown in Figure 7. Three of them are output by NN-NFFFT, while the last one Figure 7d is the result given by “Image-based” NFFFT with 401 frequency points. As we can see, the NN successfully predicts the peaks and troughs of the far-field data, and for the different TUTs, which have different numbers of scattering centers, the error does not increase.

**Figure 8:**
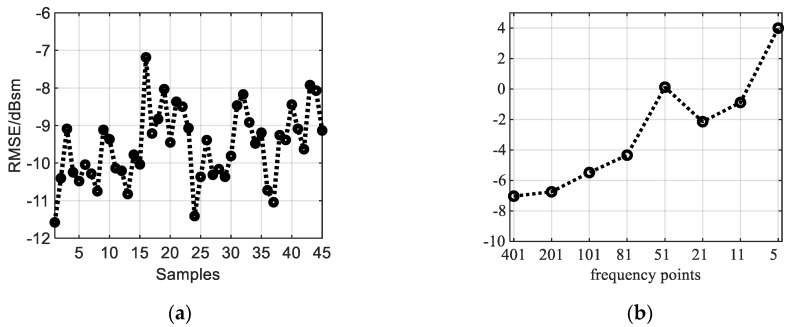
Test error (**a**) the RMSE of different sample output from neural network. (**b**) The RMSE of “image-based” NFFFT result (only the center frequency) respect to different frequency points.

The RMSE of the NN-NFFFT output RCS is shown in Figure 8a. It shows that the RMSE of the test samples is lower than −7 dBsm. Compared with the 0 dBsm targets.

The RMSE of the above mentioned “image-based” NFFFT is shown in Figure 8b. The *x*-axis stands for the different frequency points. The RMSE increases as the frequency points reduce. If the frequency points are more than 201, the RMSE is higher than −7 dBsm.

### 3.4. Run Time

As we can see, when the frequency points are lower than 201, the RMSE of “image-based” NFFFT and NN-NFFFT is at the same level. In this condition, the operation time and RMSE for each framework are shown in Table 1. The simulation platform is Intel(R) Core(TM) i7-6700 CPU @ 3.4 GHz (Intel, Santa Clara, CA, USA) with 16 GB RAM.

**Figure 9:**
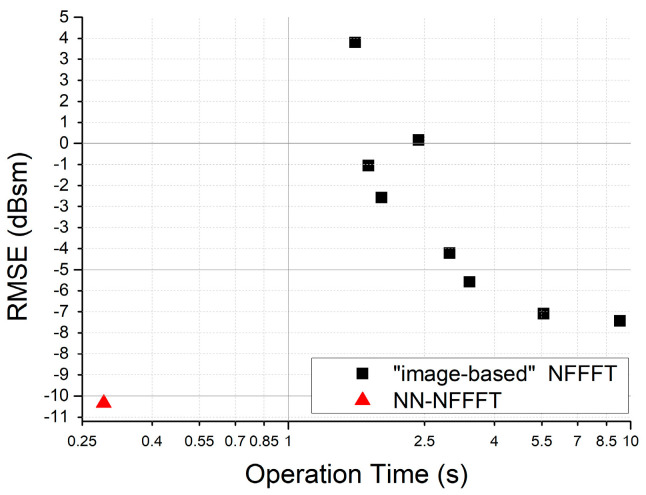
The RMSE vs. operation time of “Image-Based” NFFFT and NN-NFFFT.

The RMSE-Operation Time relationship of different NFFFT results based on the same target is shown in Figure 9. It shows that the “image-based” NFFFT needs more time to achieve the same RMSE as NN-NFFFT.

The result shows that a well-trained NN allows for real-time operation while maintaining good accuracy. Compared with the traditional “image-based” method, the framework is an alternative way to predict far-field RCS from near-field data.

### 3.5. Flexibility

The NN-NFFFT framework is proven to be an alternative approach to predict far-field RCS from near-field data. There are a few limitations in generalization due to the simple training dataset. The reason is that the framework is driven by a simple scattering model. However, the framework we proposed is flexible in multiple situations. By adjusting the training samples, the framework can be applied to more scenarios.

The first situation is that the scattering characteristics of the target and the test scenario must be considered as much as possible before training. If a new factor is encountered in the test scenario, the factor can be analyzed and considered in the training process to improve the result. This aspect is discussed in Section 4.2.

The second one is that the framework can be applied to more scattering configurations as long as the training samples are sufficient. We further apply the proposed framework to the asymmetric point scattering targets. We use double-scattering points to show the result. For more scattering center targets, the steps are the same.

The model is shown in Figure 5b. The maximum radius of the target range R is 10λ. Two scattering points are randomly distributed in the range. The distance between them is greater than 3λ to meet the near-field test conditions, and there are no other restrictions. The other test conditions remain unchanged. In this condition, TUT is asymmetric and flexibly distributed.

Based on the accuracy and the training speed, 2500 samples are chosen as the training dataset. The framework has the same structure and the same number of iterations. The training time increases to 1350 s due to the increase in samples.

**Figure 10:**
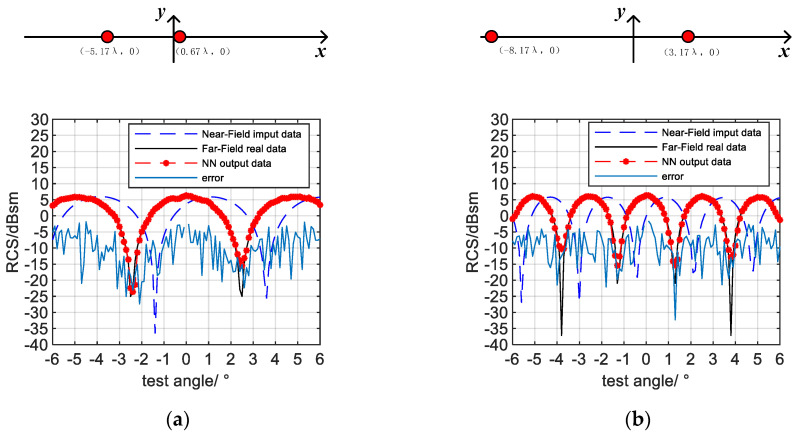
The shape and results of test targets: (**a**) asymmetric sample with little fluctuation; (**b**) asymmetric sample with fluctuation.

Two of the test samples and their results are given in Figure 10. They are randomly selected. The results show that the framework can adapt to all the two-point scatterers in the area that do not meet the far-field conditions by using a reasonable dataset.

Due to the above two factors, the framework has great flexibility, even if its generalization is limited due to the current training dataset.

## 4. Real Scene Experiment

### 4.1. Experiment Setup

**Figure 11:**
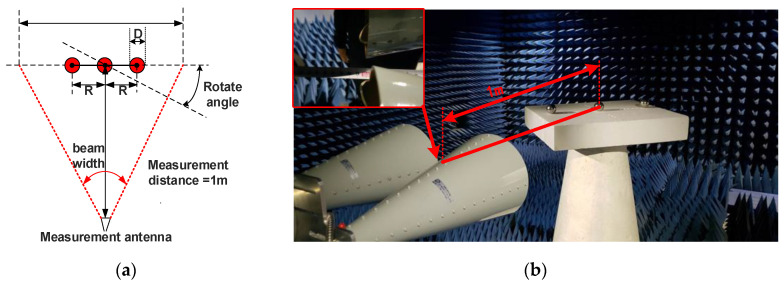
The experiment setup: (**a**) the experiment analyzes and design; (**b**) experiment environment.

In order to verify the framework on the actual scene, the experiment is carried out in an anechoic chamber using a vector network analyzer (VNA) Ceyear-3655L (China Electronics Technology Instruments Co., Qingdao, China). The experiment setup is shown in Figure 11. The measurement is carried out at 10 GHz, which has a wavelength of 3 cm. The measurement antennas are located at a distance of 1 m, which is 33λ away from the turntable center.

The aperture of the test antenna is about 0.1 m. According to antenna radiation theory [27], the antenna far-field zone is about 0.7 m. In the antenna’s far-field zone, the radiation power distribution is the same as the antenna pattern. The 3 dB beamwidth of the antenna is 20°. Thus, the aperture at 1 m away from the probe is 0.72 m, while the maximum length of the target at 10 GHz is 0.6 m. Tt means that the electromagnetic wave can cover the target completely. The setup is the same as the simulation.

Two different metal spheres are used in the experiment as different targets. According to the Mie series [1], the RCS of metal spheres is shown in Table 2, −26 dBsm, and −31 dBsm. The distance between them is more than 300 mm. In this condition, the metal sphere can be treated as a point-scattering target. The multiple reflections are much lower than the receiver’s ground noise. Thus, the effect is negligible.

### 4.2. Result and Discussion

**Figure 12:**
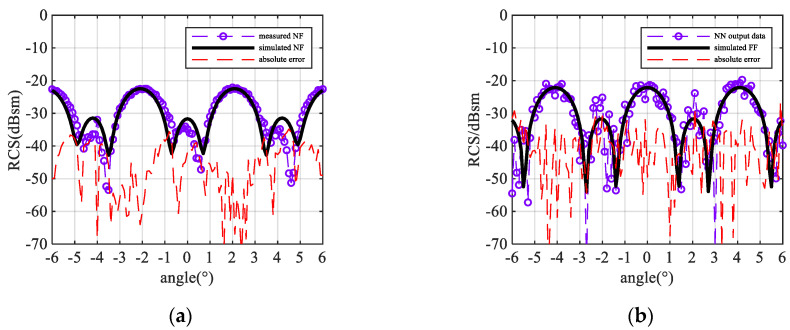
Sphere diameter = 25 mm (0.83λ), R = 0.21 m (7λ) result: (**a**) near-field measurement result and absolute error; (**b**) NN output result and absolute error.

**Figure 13:**
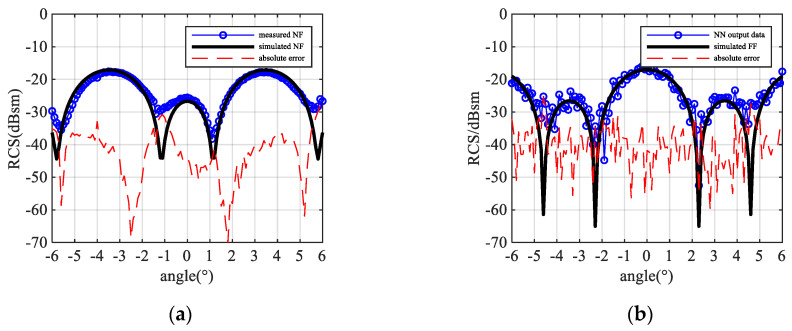
Sphere diameter = 45 mm (1.5λ), R = 0.125 m (4.16λ) result: (**a**) near-field measurement result and absolute error; (**b**) NN output result and absolute error.

**Figure 14:**
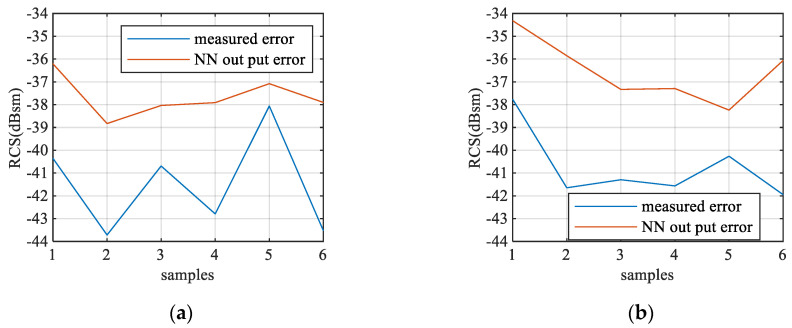
Measurement and neural network error (NN without optimization): (**a**) RMSE of each sample for 25 mm (0.83λ) diameter sphere; (**b**) RMSE of each sample for 45 mm (1.5λ) diameter sphere.

Six groups of experiment samples are randomly selected, and each group uses two different metal spheres in Table 2. Two of the measurement result and output RCS predicted by the framework are shown in Figure 12 and Figure 13. By using the 50 Hz IF bandwidth, the absolute measurement error is lower than −38 dBsm. The NN predicts the peaks and troughs of the far-field. All groups’ measurement RMSE and prediction RMSE are shown in Figure 14. The RMSE of the predicted far-field data remains lower than −34 dBsm and −36 dBsm as the target’s RCS becomes larger.

As shown in Figure 12 and Figure 13, the RCS, predicted by NN, jitters compared with the reference result. This is because of the noise introduced by the test equipment. The signal to noise ratio (SNR) can be approximately calculated by

(23) $$\mathrm{SNR}=\frac{\mathrm{signal}\;\mathrm{power}}{\mathrm{noise}\;\mathrm{power}}=10\log\frac{S_{0}{}^{2}}{{\left(S-S_{0}\right)}^{2}}$$

where *S*_0_ is the absolute value of the signal (obtained by simulation), and *S* is the actual value measured by sensors. The SNR of the measurement result is 14.5 dB for the 25 mm sphere and 17 dB for the 45 mm sphere. The SNR of the NN output result is 10.5 dB for the 25 mm sphere and 13.1 dB for the 45 mm sphere. The SNR deteriorates by 4 dB, and the added noise mainly comes from the framework.

In order to evaluate the impact of noise, the VNA’s noise needs to be analyzed. The received power can be calculated by radar function

(24) $$P_{r}=\frac{P_{t}G_{t}\sigma G_{r}\lambda^{2}}{L_{t}{\left(4\pi \right)}^{3}r^{4}L_{r}}$$

where Pt is transmitted power, it is equal to −30 dBm. *G_t_* and *G_r_* stand for the antenna gain, which are around 13 dBi. The other parameters are mentioned in the previous sections. The loss *L_t_* and *L_r_* are unknown. Thus, the received power is about −130 to −140 dBm.

The receiver sensitivity is about −150 dBm. It can be calculated by the function kTBF, where k is the Boltzmann constant. T is the Kelvin temperature, which is 300 K. B is the IF bandwidth, which is 50 Hz. F is the noise figure of the receiver, which is 6 dB. The SNR of the measurement result is around 10 to 20 dB. It is the same as the SNR calculated by Equation (23).

This is the reason why fluctuation exists in the results. The noise is not considered in the training process. Thus, the trained NN treated noise as a useful signal while processing the measurement data.

The noise needs to be considered in the training process to reduce its impact on the output results. The NN is a flexible denoising framework in signal processing [28] as long as the noise is considered in the training dataset. It can reduce the noise by merely replacing the noisy signal to the training dataset. The Gaussian white noise is added into the near-field data, and then the noisy near-field data and noise-free far-field data constitute new data pairs. They are added to the training dataset. The SNR of the near-field is 15 dB. The training samples is increased to 1344, while the training time is expanding to 566 s.

**Figure 15:**
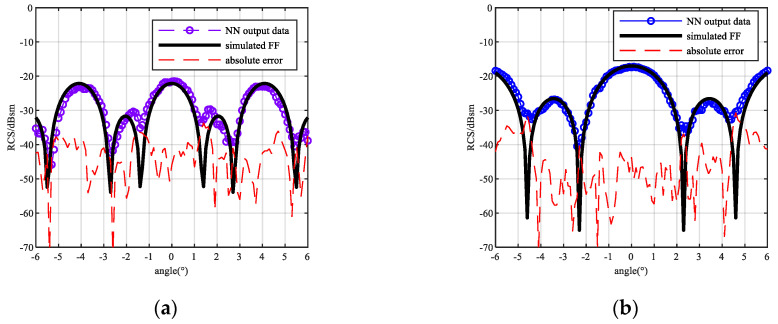

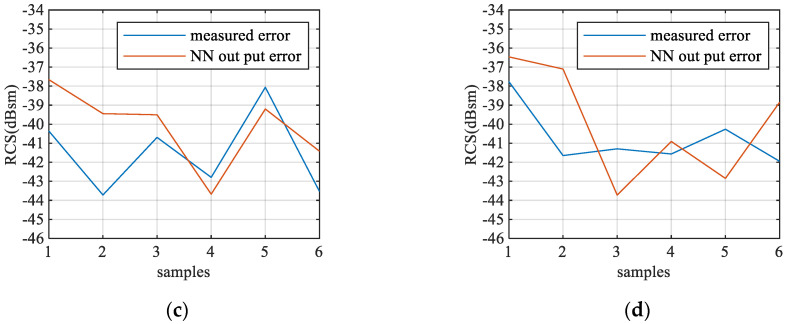
Result and RMSE output by improved NN: (**a**) Sphere diameter = 25 mm (0.83λ), R = 0.21 m (7λ) NN output result; (**b**) Sphere diameter = 45 mm (1.5λ) R = 0.125 m (4.16λ) NN output result; (**c**) RMSE of each sample for 25 mm (1.5λ) diameter sphere; (**d**) RMSE of each sample for 45 mm (0.83λ) diameter sphere.

By changing the training dataset, the framework is used to the same measurement near-field data. The result is shown in Figure 15.

The improved framework successfully predicted the far-field picks and troughs, and the output RMSE of the data is reduced to the same magnitude as the measurement data. The SNR of the result is 14.42 dB for the 25 mm sphere and 17.4 dB for the 45 mm sphere. It shows that the SNR improves and is equal to the input one.

The result proves that the NN-NFFFT framework is highly adaptable. The accuracy of the framework mainly depends on the selection of the training dataset. If various situations in practical applications are considered during the training process, the framework’s ability in practice will also be powerful.

Furthermore, the measured data are single frequency scatter data. Thus, the “image-based” NFFFT cannot be used. If a broadband measurement is carried out, it takes a long time in data acquisition. In the above experiment, the RCS of the TUT is −26 dBsm and −31 dBsm, which is very small. It requires a relatively small IF bandwidth in VNA. Thus, the IF bandwidth is 50 Hz, and the testing point in the angle domain is 121. The speed of the turntable is 1 degree per second. The data used in the abovementioned framework can be acquired in about 12 s (= 12 ÷ 1). Correspondingly, if a broadband measurement with 101 points in the frequency domain is implemented, the measurement time is around 244.5 s (101 ÷ 50 × 121). It shows that a single frequency measurement is much more efficient in the data acquisition process. The experiment shows that the framework has potential in RCS NFFFT.

## 5. Conclusions

In this paper, a flexible Neural Networks Near-Field to Far-Filed Transformation (NN-NFFFT) framework has been proposed to predict far-field RCS from near-field RCS. This framework contains three significant steps. The first one is making a dataset. Targets in the dataset are based on the actual demand, and the shape of the measurement scatterer should fit the same distribution. In our framework, point-scattering targets conforming to the Swerling Case I Model are used to simulate the dataset. The second one is training the regression estimator. The accuracy of the framework depends on this process and the time consumption is mainly concentrated in this process. The last one is the prediction of far-field RCS. The result proved that NN-FFFFT is a potential method to achieve NFFFT, while for the specific TUT, the far-field RCS can be predicted by a neural network with efficient priori information. What is more, the framework increases operation speed while maintaining acceptable accuracy compared with the widely used “image-based” NFFFT method. The measured near-field data have been used in the framework. The result shows that the framework has scalability and can improve performance further by employing prior information about the measurement scenario. Furthermore, during the actual data acquisition, the method maintains good accuracy with less time.

## Figures and Tables

**Table 1:** Temporal comparison.

Framework	Pre-Preparation Time ^1^	Operation Time	RMSE	Experiment Data Acquisition Time ^2^
NN-NFFFT	275 s	0.289 s	−10.28 dBsm	12 s
“image-based” NFFFT	none	5.568 s	−6.748 dBsm	244.5 s

^1^ The pre-preparation time means the time consumption before the framework’s implementation, and it mainly refers to the training time of the neural network. ^2^ The experiment data acquisition time is explained in the Experimental Results section. It is estimated under the following conditions: the VNA IF bandwidth is 50 Hz, the testing point in angle domain is 121 and the speed of the turn table is 1 degree per second.

**Table 2:** Measurement target.

Sample Number	Metal Sphere Diameter	Project Area of the Sphere	NRCS ^1^ a at 10 GHz	RCS
1	45 mm	−27.987 dBsm	1.427 dB	−26.557 dBsm
2	25 mm	−33.092 dBsm	1.433 dB	−31.665 dBsm

^1^ NRCS stands for the normalized radar cross section for perfectly conducting sphere respect to the projected area. NRCS = σ/πa^2^, where σ stands for the RCS of sphere, and a represents the radius.
